# PV-Assisted grid connected multi output electric vehicle charger with PV2V, G2V and PV2G functions

**DOI:** 10.1371/journal.pone.0304637

**Published:** 2024-06-21

**Authors:** Ramanathan G., Bharatiraja Chokkalingam, J. L. Munda

**Affiliations:** 1 Centre for Electric Mobility, Department of Electrical and Electronics Engineering, SRM Institute of Science and Technology, Kattankulathur, Chennai, India; 2 Tshwane University of Technology (TUT), Pretoria, South Africa; University of Cagliari, ITALY

## Abstract

The demand for renewable energy-based Electric Vehicle (EV) charging infrastructure is increasing in recent years. Solar PV based EV charging method is preferred as it has simple energy harvesting technique. The PV system is an uncertain power source, where the power generation is varied with respect to the availability of sunlight. So, that the charging station requires a backup power supply for the uninterrupted charging. For the integrated power sources, the charging station requires a simple and efficient conversion unit for the DC/AC/DC conversion. In this work, a modified Z-source inverter (MZSI) is developed for the multiport EV charger using PV and grid. The proposed MZSI is connected between the input and output sides to boost the voltage as per the demand at the battery side. In order to connect many battery units with the charger, the capacitors used in the MZSI are split as per the required number of charging ports. This developed converter topology operates the systems in four different modes like PV-Grid, PV-battery, grid-battery, and battery-grid. The performance of this proposed work has been validated in *MATLAB/Simulink®* and in the experimental setup. The experimental setup has been developed with two charging ports for obtaining 250W at each charger end which cumulatively produces 500W output across both chargers with an efficiency of 90.18%.

## I. Introduction

The commercialization of electric vehicles has accelerated in recent years due to their numerous advantages [1, 2]. Charging stations are a critical component of EV technology, and charging methods and charger technology vary depending on the type of application. For long-distance applications, EVs require greater battery backup and faster charging methods. There are three types of charging methods: on-board charging, off-board charging, and wireless charging. These can be further classified into two categories: fast charging and slow charging [3]. EV charging methods require converter topologies for DC/AC, DC/DC, and AC/DC conversion with minimal power loss [4–6].

EV charging station PV systems reduce operational costs, promote sustainability, and strengthen grid resilience [7, 8]. Solar PV cuts fossil fuel and EV charging emissions. Integration reduces peak demand grid dependence and controls charging station operators’ electricity costs. Multiple power losses make PV system installation difficult. PV panel inverters lose efficiency, shading and soiling reduce panel effectiveness, temperature sensitivity reduces efficiency under high heat, and irregular panel manufacturing and electrical connections cause mismatch and wiring losses 8. There are different solutions are available such as maximum power point tracking [9], array configuration [10, 11], array reconfiguration [12] and so on. Grid integration is needed for energy management despite these issues. Excess energy is fed back into the grid and used to generate credits or revenue to offset overproduction and deficits. Grid connectivity supports smart grid technologies and power during low solar output. These technologies optimise grid stability and energy efficiency by optimising electricity production, distribution, and consumption [13]. The converter topology for the DC-AC conversion in bidirectional is an important factor for the efficient operation in the EV charging stations. There are different converter topologies specifically for the EV applications are developed previously. EV charger was developed using CUK arrangement-based isolated converter. In this converter topology operation, at zero Voltage Source mode that eliminates switching loss. Negative output can obtained using the CUK converter [14]. The charging time and waiting time are some of the constraints considered at the charging station [15]. An optimization problem based multiport charging stations can be the better solution for reducing the above constraints [16–18]. The multiport charging stations are become famous in recent times due to this reasons. These station requires more power back-up for the continuous power supply. The grid based multiport charging stations can overcome the power back-up requirement. There will be more fluctuations on voltage, power and power quality issues in renewable energy based charging stations. In order to avoid these problems, grid integrated charging stations are preferred [19]. There are many problems like voltage fluctuations will rise on the grid integration process. For reducing the losses and other disturbances, different types of converters are used. The basic concept in grid integration is to get charge from the renewable sources, when there is sufficient generation to meet out the demand. When the generation is not sufficient, the charging station need to get power from the grid and when the generation is more than the demand, it supplies the power to grid [20]. In recent years, there has been a greater emphasis on PV-based charging infrastructure for EVs [21]. Off-board charger with battery backup and grid supply allows quick charging. As it requires a rectifier circuit that limits the bidirectional power flow. For grid integration, the voltage of the source should be matched with the grid voltage, which can be achieved by using the ZSI [22]. Due to the existence of two capacitors, the voltage in ZSI is doubled. In [23], the performance of the capacitor and inductor in the ZSI is analyzed.

Due to the lack of an isolation transformer, a fault on the supply side will damage the load. At the same time, placing the transformer in between the load and supply creates galvanic isolation [24]. In order to eliminates power fluctuations and avoids the fault occurrence on the load side, bidirectional converters with various topologies were developed. The concept of bidirectional converter is to deliver power to the load and consume power from the load. There are many bidirectional converters are used for this kind of applications. The construction and different modes of basic bidirectional converters are discussed [25, 26]. The conventional converters are integrated with model predictive control to find an appropriate power flow trajectory to improve energy resiliency, minimize operational costs, and optimize micro grid profit. An H-bridge inverter is developed for polarizing the different voltage states. The switches used in this converter allows higher voltage level, it reduces the grid current and total harmonic distortion. This inverter has better efficiency due to the low switch count and minimum switching loss.

Dual active bridge (DAB) converters are popular choices for electric vehicle (EV) charging applications [27]. However, the selection of the converter topology also depends on specific design requirements, such as power rating, efficiency, cost, and control complexity [28]. By incorporating the demand response as the reference signal for calculating the duty cycle, the switching losses and thermal stress can be reduced. This improves the performance and life span of the inverter [29, 30]. The power flow between the PV and grid are the important factors that needs to be considered in the PV-grid tied system [31, 32]. Dual in dual out converters (DIDO) also plays a kind of solution for the efficient power flow between sources and charging stations [33, 34]. Z- Source Inverter (ZSI) topology uses an impedance network to achieve voltage boosting and bucking, making it suitable for applications that require high voltage gain. The Z-Source inverter can also operate in a bidirectional mode, which allows it to transfer energy between the battery and the grid. It has many advantages over DAB converters and other converter topologies. The DC-AC conversion process and multiport charging options are the main drawbacks of ZSI. These problems can be overcome by the proper modeling of ZSI. In the quasi-Z-source inverter, a unique capacitor-inductor impedance network connects the converter main circuit to the power source. This design allows single-stage voltage buck and boost without DC-DC converters. QZSI is efficient and compact, making it useful in applications that need a wide input voltage range and robust power conditioning [35, 36]. In photovoltaic (PV) grid integration, qZSIs manage solar input fluctuations and maintain output voltage compliance. More reliable and efficient solar energy transfer to the grid [37]. EV charging is fast and efficient with qZSIs, which adapt to battery voltages and charging standards. For dynamic EV charging stations, qZSIs can efficiently manage high power and varied load conditions [38].

GaN technology and an interleaved iL2C DC-DC converter are used to create a high-efficiency electric vehicle charger with 98.2% efficiency over a wide input voltage range [39, 40]. However, the system’s complexity, cost, scalability, and thermal management may affect deployment and reliability. A 3.3 kW iL2C resonant full bridge converter for xEV charging was parametrically modeled [41]. Power density and load distribution are improved by hybrid variable frequency and phase shift modulation control. For dynamic electric vehicle charging scenarios, steady-state and transient simulations at various input voltages show strong performance.

**Fig 1:**
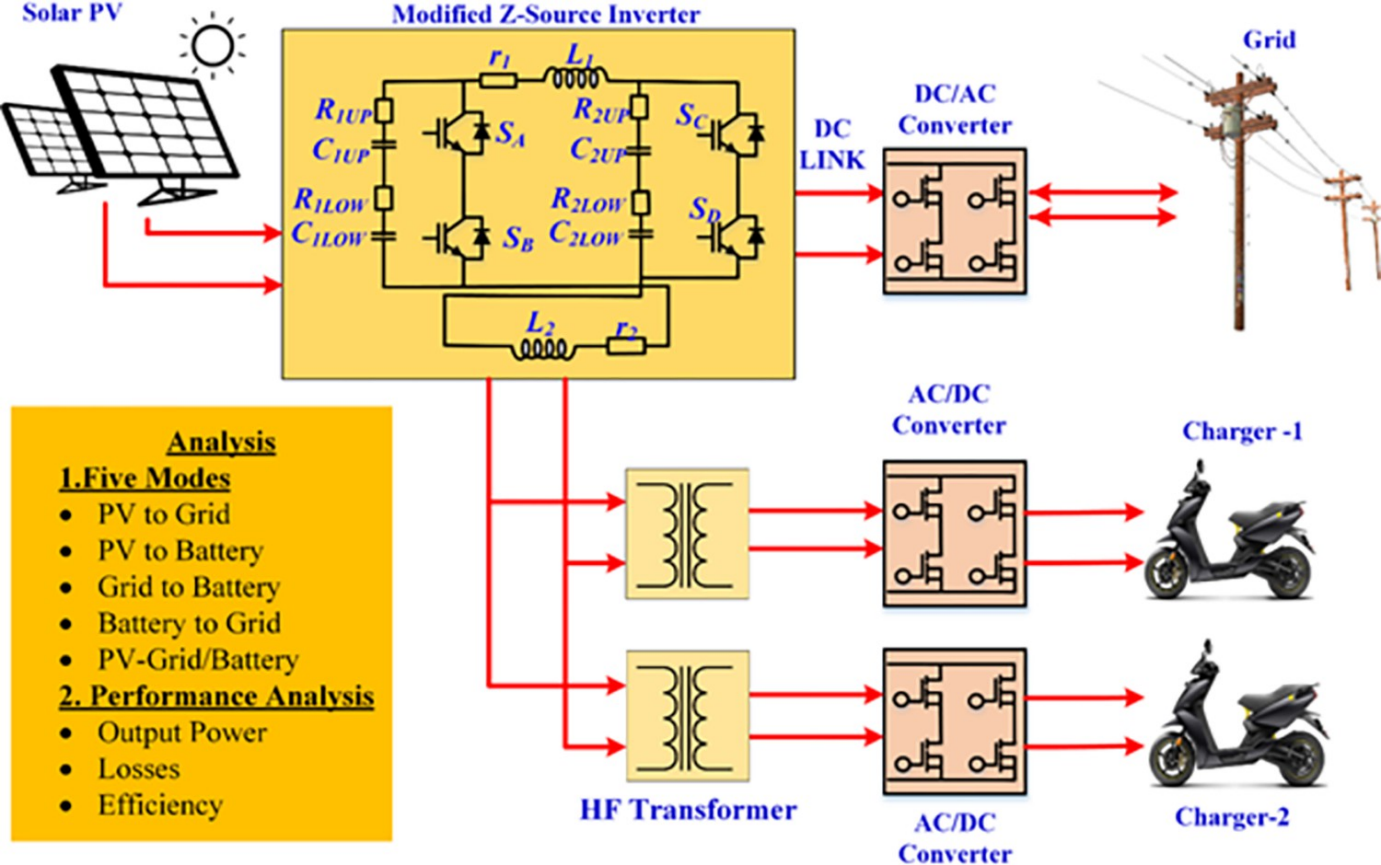
Functional block diagram of the proposed topology.

This work proposes the modified Z Source Inverter (MZSI) for achieving this objectives. By modifying the construction of ZSI, the multi ports can be created on single inverter which has been developed with necessary mathematical expressions. The performance of the proposed work is validated in four different modes operation using simulation and experimental setup. The simulation analysis is performed on *MATLAB/Simulink®* and the experimental setup is developed with two charging ports for obtaining 250W at each charger end which cumulatively produces 500W. The functional block diagram of the proposed system is shown in Fig 1. The structure of the proposed work is as follows: section 2 describes the fundamental concept of Z-Source Inverter and modeling of the proposed converter topology and sizing. The performance analysis of the proposed work using simulation and experimental was presented in section 3 and section 4. The inferences and conclusions from the analysis are presented as the conclusion in section 5.

## 2. Proposed Modified Z-Source Inverter (MZSI)

The ZSI has four switches, two capacitors, and two inductors for the operation. This inverter can generate buck or boost output as per the duty cycle. Also, shoot-through and non-shoot-through operations can be performed using this inverter topology.

The current across the inductors and voltage across the capacitors can be expressed as Eqs (1) and (2) as follows.


$$i_{L}=i_{L1}=i_{L2}$$ (1)

$$V_{C}=V_{C1}=V_{C2}$$ (2)

The ST state is determined by the switching frequency F_SW_ and the duty cycle D_O_. The voltage across the capacitors is expressed as Eq(3)

$$V_{C}=\frac{1-D_{O}}{1-2D_{O}}V_{PV}$$ (3)

The dc-link voltage V_PN_ can be expressed as Eq (4),

$$=\frac{1}{1-2D_{O}}V_{PV}$$ (4)

The power ratio between the ac and dc sides of the ZSI is expressed as

$$(1-D_{O}{)V}_{PN}I_{PN}=v_{grms}i_{grms}$$ (5)

Where V_PV_ PV voltage, *V*_*PN*_ dc-link voltage, I_PN_ dc-link current modulation index is represented by M.

The Grid voltage is assumed as Eq(6),

$$V_{g}=MV_{PN}$$ (6)

The ZSI’s RMS output voltage can be expressed as Eq (7)

$$V_{grms}=\frac{MV_{PV}}{\surd 2(1-2D_{O})}$$ (7)

**Fig 2:**
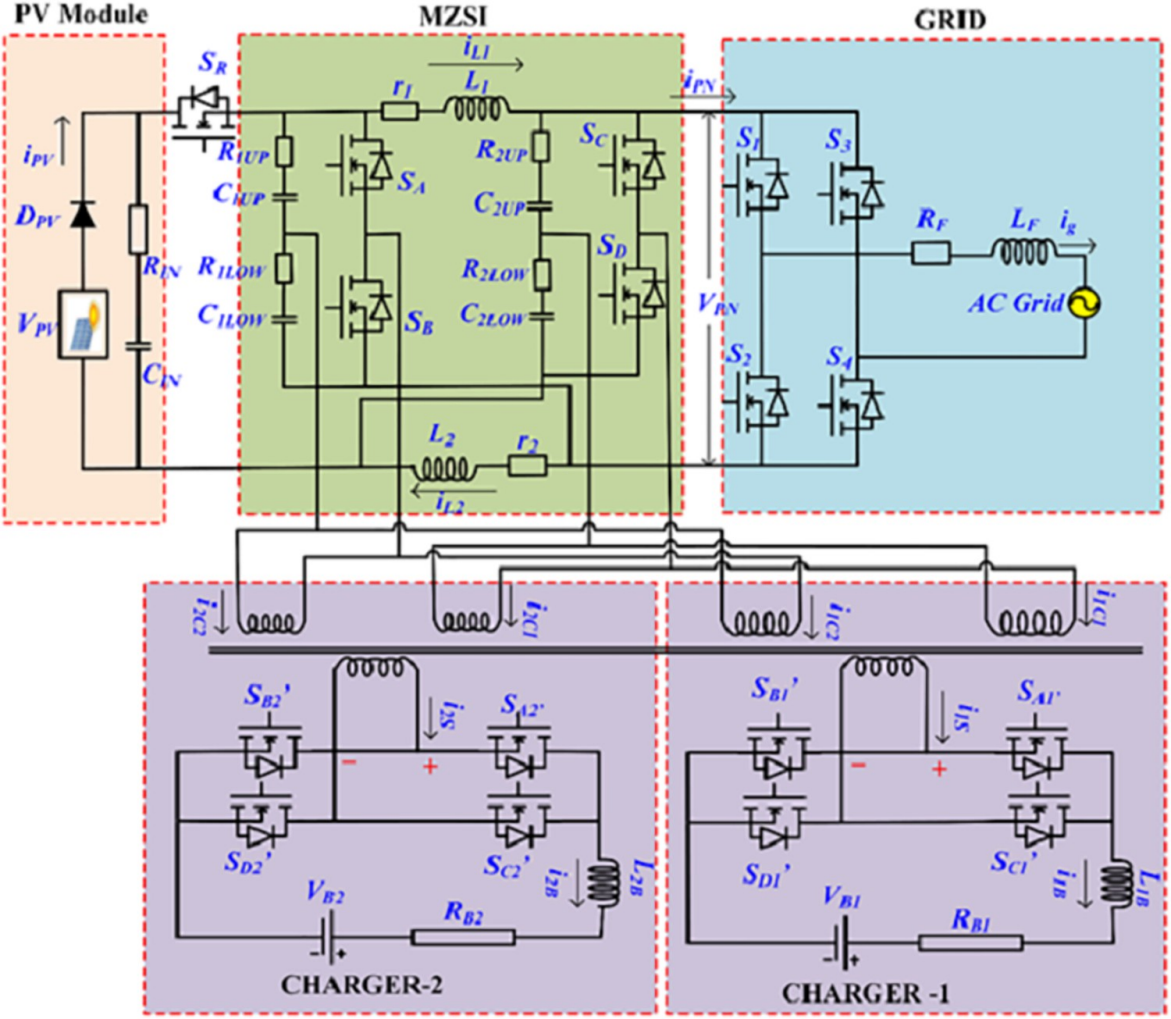
Circuit diagram of the proposed MZSI.

In this proposed work, the conventional Z source inverter is modified for creating a multiport EV charger. In conventional topology, the inverter has two capacitors C_1_ and C_2_. These two capacitors are split into two equal-rated capacitors to divide voltage. The circuit diagram and the function of the proposed MZI for the multiport charging environment are shown in Fig 2. The normal capacitors C_1_ and C_2_ in the conventional ZSI are split into two capacitors like C_1UP_, C_1LOW_, C_2UP,_ and C_2LOW_. The charging station is operated with two power sources Solar PV system and Grid integration. The solar PV system supplies the required demand for the charging station. When the demand at the charging station cannot meet out by the PV, the charger takes supply from the grid. The grid compensates for the shortage of power demand. When the PV generates power more than the demand, then the surplus power will be delivered to the grid. In another scenario, the grid needs to meet the entire demand at the charging station during the unavailability of sunlight. This power environment is completely uncertain because of the uncertain load and uncertain generation from PV. The conventional ZSI can handle this scenario, but multiport tapping cannot be achieved. In this work, the modified inverter topology can manage this uncertain power flow and regulates the constant voltage at the charger terminals. Three high-frequency transformers were used for changing the voltage level between the grid to MZSI, MZSI to the grid, MZSI to the battery, and the battery to MZSI. Along with these transformers, H-Bridge Converter (HBC) have been used for regulating the voltage and isolating the sources from the grid.

In the MZSI, the voltage across the capacitor is double the value of the battery voltage and it can be expressed as Eq (8),

$$V_{C}=2V_{B}$$ (8)

Where, V_B_ is the battery voltages, V_C_ is the capacitor voltage. The duty ratio for the ST operation can be obtained from the battery voltage and PV input and it can be calculated from the Eq (9),

$$D_{Omax}=\frac{2V_{Bmax}-V_{pvmin}}{4V_{Bmax}-V_{pvmin}}$$ (9)

The inductor values can be obtained for the specific inverter using maximum capacitor voltage (V_Cmax_), maximum ST duty ratio, and frequency current ripple. The expression for calculating the inductance value is given in Eq (10),

$$L_{1}=L_{2}=\frac{V_{Cmax}D_{Omax}}{2\Delta i_{L}f}$$ (10)

Where, D_omax_ is the maximum ST duty ratio, L_1_ is the inductor 1 value, L_2_ is the inductor 2 value. The capacitors were designed in order to reduce the second-order harmonics in the capacitor voltages. Along with this an L filter is also been used to reduce the complexity on capacitor ratings. The expression for designing the capacitance can be expressed as Eq(11),

$${C_{1UP}=C_{1LOW}=C_{2UP}=C_{2LOW}=2C}_{1}={2C}_{2}=\frac{2P}{2\omega \Delta V_{C}V_{C}}$$ (11)

Where the VC is the voltage across the capacitors and it can be represented as Eq (12)

$$V_{C}=\frac{V_{C1}+V_{C2}}{2}$$ (12)

The voltage across the each splitter capacitors are given in Eqs (13–14),

$$V_{C1_{UP}}=V_{C1_{LOW}}=\frac{V_{C1}}{2}$$ (13)

$$V_{C2_{UP}}=V_{C2_{LOW}}=\frac{V_{C2}}{2}$$ (14)

Where, D_omax_ is the maximum ST duty ratio, L_1_ is the inductor 1 value, L_2_ is the inductor 2 value, C_1UP_ is the first half of C_1_ capacitor, C_1LOW_ is the second half of C_1_ capacitor, C_2UP_ is the first half of C_2_ capacitor, C_2LOW_ is the second half of C_2_ capacitor.

One H-bridge converter is connected between MZSI and the grid. This regulates the voltage between the inverter and the grid. Two sets of H-bridge converters are used in between batteries and the MZSI. The modified multiport Z source inverter is connected between the PV module, grid, and battery. It can operate in four different modes based on the requirement. The modes are, Mode-I (PV to Grid), Mode-II (PV to the battery), Mode-III (Grid to Battery), and, Mode-IV (Battery to Grid). For each mode of operation, some of the switches need to turn OFF and some of the switches need to be turned ON. The switches in MZSI, H-bridge converters. Charger 1, and, charger 2 are regulated for operating the proposed work in five different modes.

In mode 1, PV supplies power to grid, where MZSI switches and HBC1 switches are turned ON. In mode 2 PV supplies power to the battery and in this conditions HBC1 is turned OFF and HBC2 and HBC3 connected with chargers has been turned ON. In mode 3 and mode 4, power has been delivered to battery from grid and to grid from battery where in these modes MZSI converters and all HBC switches are turned ON. The switching operating conditions on different modes are given in Table 1.

**Table 1:** Switching operations on different modes.

Modes		MZSI (S_A_,S_B_,S_C_,S_D_)	HBC1 (S_1_,S_2_,S_3_,S_4_) (GRID)	HBC2 (S_A1’_,S_B1’_,S_C1’_,S_D1’_) (Charger 1)	HBC3 (S_A2’_,S_B2’_,S_C2’_,S_D2’_) (Charger 2)
Mode-I	PV-Grid	ON	ON	OFF	OFF
Mode-II	PV-Battery	ON	OFF	ON	ON
Mode-III	Grid-Battery	ON	ON	ON	ON
Mode-IV	Battery-Grid	ON	ON	ON	ON
Mode-V	PV-Grid/Battery	ON	ON	ON	ON

### 2.1 Mode—I: PV to grid

**Fig 3:**
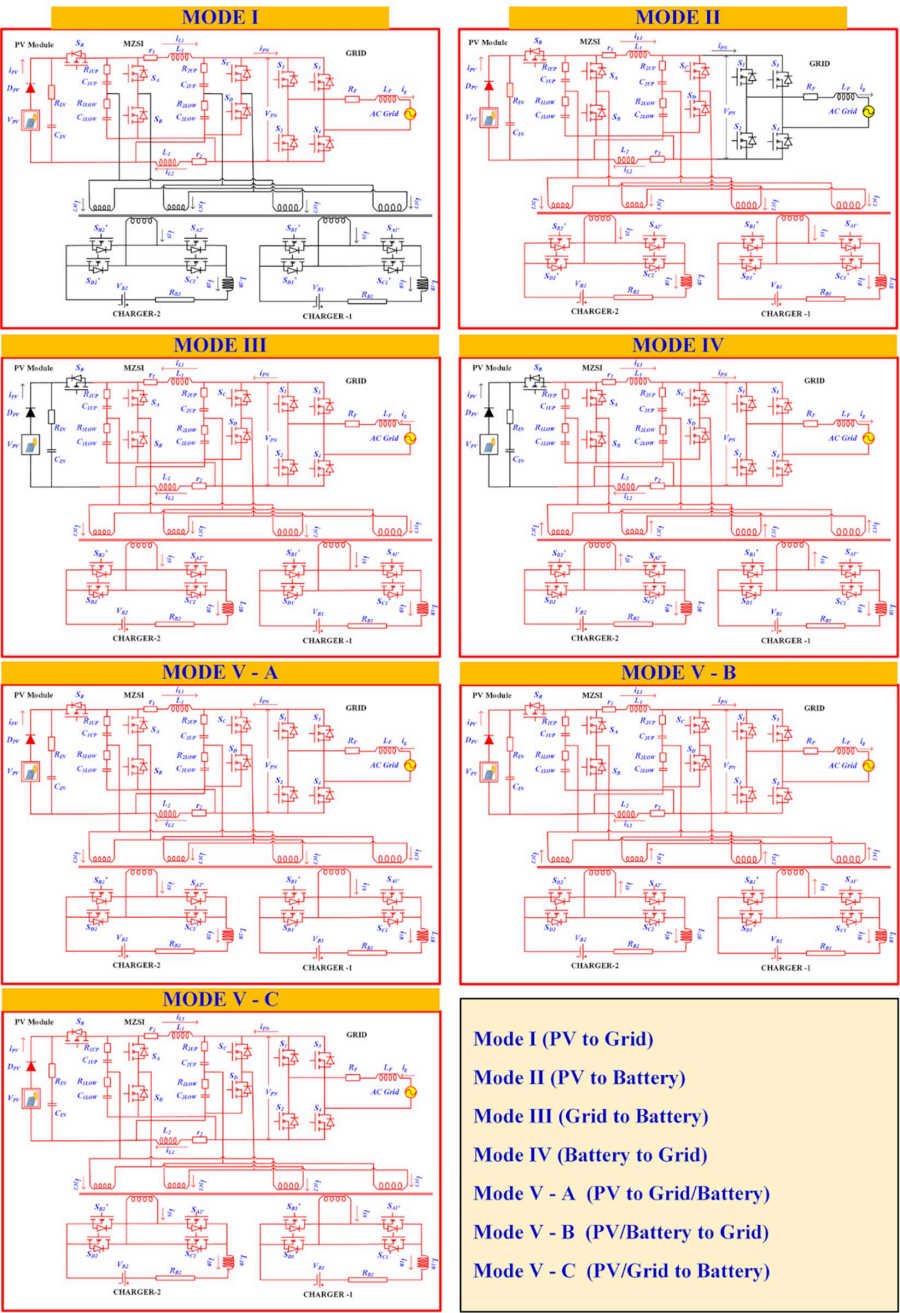
Various modes of operation.

In this mode, the PV system supplies the power to the grid where the MZSI is acts as normal inverter. The power will flow to the grid through MZSI and HBC-1. In this mode, S_1_, S_2_, S_3_, S_4_ switches and diodes in HBC need to be turned ON and turned OFF for 50% of duty cycle. S_1_, S_4_ switches are turned ON for the positive half cycle and S_2_, S_3_ switches are turned ON for the negative half cycle. The duty cycle for each switch is 0.5. The PV system generates DC supply that needs to be converted into AC. So that the supply can be synced with the grid. The switches S_A_, S_B_, S_C_ and S_D_ are operated with the 25% of duty cycle for the conversion of DC to AC. Fig 3 shows the circuit diagram of the proposed MZSI where the red lines are indicates the active region and black lines indicates the non-active regions.

### 2.2 Mode—II PV to battery

In mode II, the PV system supplies the power to the battery and in this mode the MZSI is acts as normal inverter. The power will flow to the battery through MZSI and HBC-2 and HBC-3. In this mode, S_A1_, S_B1_, S_C1_, S_D1_ switches in HBC-2 and S_A2_, S_B2_, S_C2_, S_D2_ switches in HBC-2 need to be turned ON and turned OFF with 50% of duty cycle. S_A1_, S_D1_, S_A2_, S_D2_, switches in both HBC 1 and HBC 2 are turned ON for the positive half cycle and S_B1_, S_C1_, S_B2_, S_C2_, switches in both HBC 1 and HBC 2 are turned OFF for the negative half cycle. Each switches are operating with the 50% of duty cycle. The AC output from the MZSI is fed to the HBC converter for the AC-DC conversion. After the conversion, the DC supply can be stored in battery or it will charges the batteries in EV through the charging port. Fig 3 shows the circuit diagram of the proposed MZSI where the red lines are indicates the active region and black lines indicates the non-active regions.

### 2.3 Mode—III grid to battery

Mode III establishes the connection between the battery and the grid. During the unavailability of PV, the charging station requires power for charging the vehicles. In this mode, the grid will supply the entire power to the charging station. The proposed MZSI regulates the supply between the grid and the battery. The duty cycle is been varied with respect to the demand. In this mode, all three H-bridge converters are operating where each switch operated with 50% of the duty cycle. Fig 3 shows the circuit diagram of the proposed MZSI where the red lines are indicates the active region and black lines indicates the non-active regions.

### 2.4 Mode—IV battery to grid

Mode IV establishes the connection between the battery and the grid. In this mode, the battery can support the grid on its demand. The proposed MZSI regulates the supply between the grid and the battery. The duty cycle is been varied with respect to the grid demand. In this mode, all three H-bridge converters are operating where each switch operated with 50% of the duty cycle. Fig 3 shows the circuit diagram of the proposed MZSI where the red lines are indicates the active region and black lines indicates the non-active regions.

### 2.5 Mode—V PV to grid/battery

In this mode of operation, all three sources are connected with the proposed MZSI. In mode V (A), the PV system supplies the power to the grid and battery where the PV generates the rated power and able to manage the load at charger side and grid side. The PV power generation is not consistent for the whole day and it cannot support the battery and grid all the time. When the PV is completely shut down, the power will flow between grid to battery or battery to grid as discussed in mode III and mode IV.

**Fig 4:**
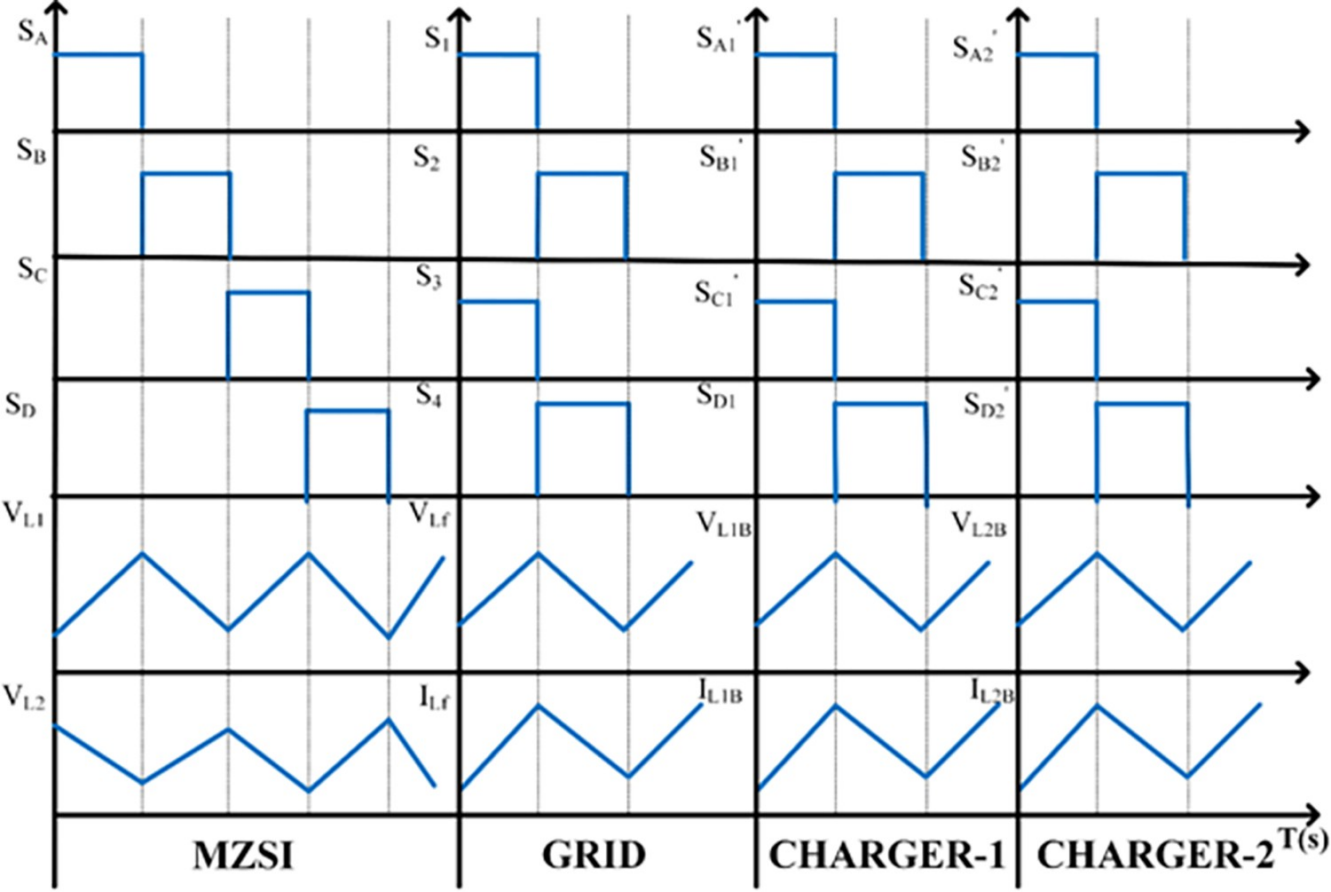
Switching operations on different modes.

There are some other possibilities are there such as the PV generates less power than the required power demand at grid side/battery side. In this mode, there are two sub modes may be possible such as the PV cannot able to supplies the demand at battery side where the grid supports with PV to manage the demand at battery side. In another condition, the MZSI can able to regulate the battery power to support the grid, when the grid experiences more stress to manage the load demand, and the PV generation is not sufficient to handle and support it. The PV generation rate has been varied to validate this different scenario. The corresponding switching operations on each mode has been represented as waveform as shown in Fig 4.

For the one complete cycle, the average current in the transformer is zero. Whereas the average current entering the switch is 25% of the I_LB_. The average current became zero due to the sharing current at all primary sides and duty cycle. Due to this, the voltage across each transformer became 50% of the capacitor voltage (V_C_) This 50% of V_C_ can be said as the average recovered secondary voltage. This made to construct the equivalent circuit as a simple model. The H-bridge AC-DC converters and the batteries are connected with the capacitors as a parallel branch which is taped using the high frequency transformers. There are four switches between H-bridge converters, and the switches are turned ON for 50% of the total time and turned OFF for 50% of the total time. On these operating conditions the inductance value will be L_B_ and the capacitance value will be C_HB_. The corresponding capacitance value and parallel branch inductance value can be expressed as the Eqs (15)–(18).


$$C_{HB}V_{HB}=i_{L}-i_{IN}-\frac{1}{4}i_{B}$$ (15)

$$L_{B}i_{B}=V_{HB}(i_{L}i_{IN})R_{HB}\frac{1}{2}(R_{HB}+{2R}_{B})i_{B}-V_{B}$$ (16)

$$C=\frac{1}{2}C_{HB}$$ (17)

$$r_{c}=\frac{1}{2}R_{HB}$$ (18)

## 3. Simulation results and analysis

**Fig 5:**
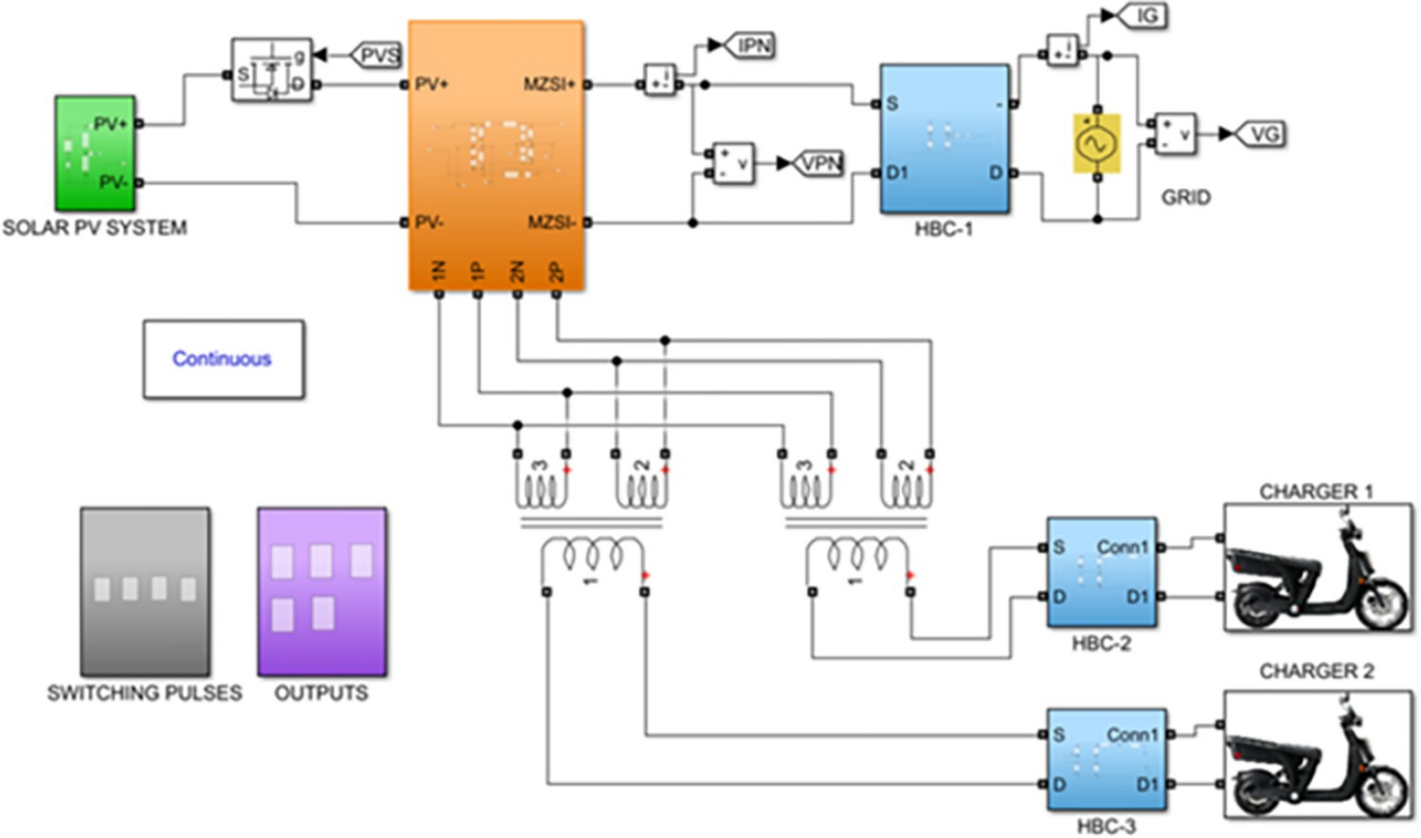
Simulation model of the proposed MZSI.

The proposed inverter topology is been built and modelled in MATLAB/Simulink® software as shown in Fig 5. A controlled DC source is been used as the PV source for the proposed work. The maximum output voltage from the solar PV panel 240V DC. This voltage is fed to the MZSI block. In simulation, the Z source inverter is constructed using the MOSFET switches. The switching pulses are generating using the PWM technique with the switching frequency of 25 kHz.

**Fig 6:**
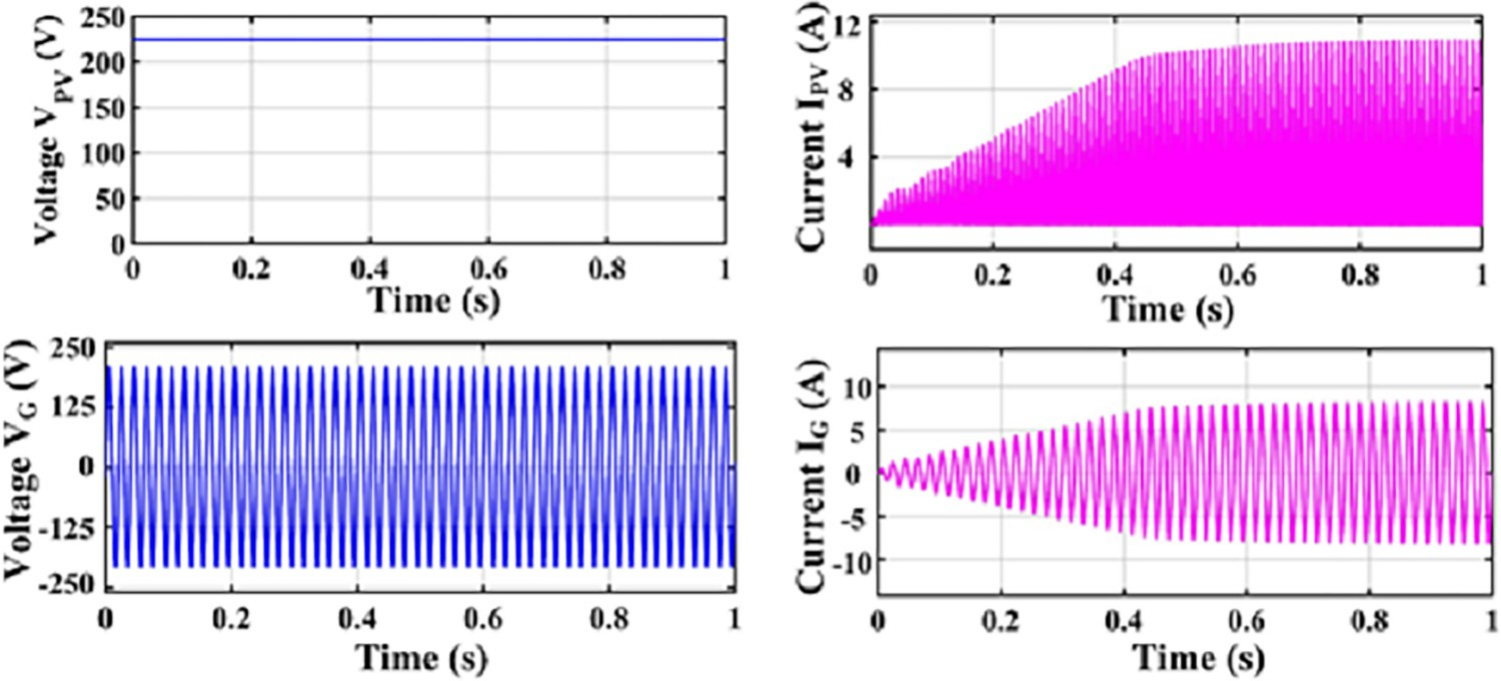
Mode I–PV to grid output waveforms.

**Fig 7:**
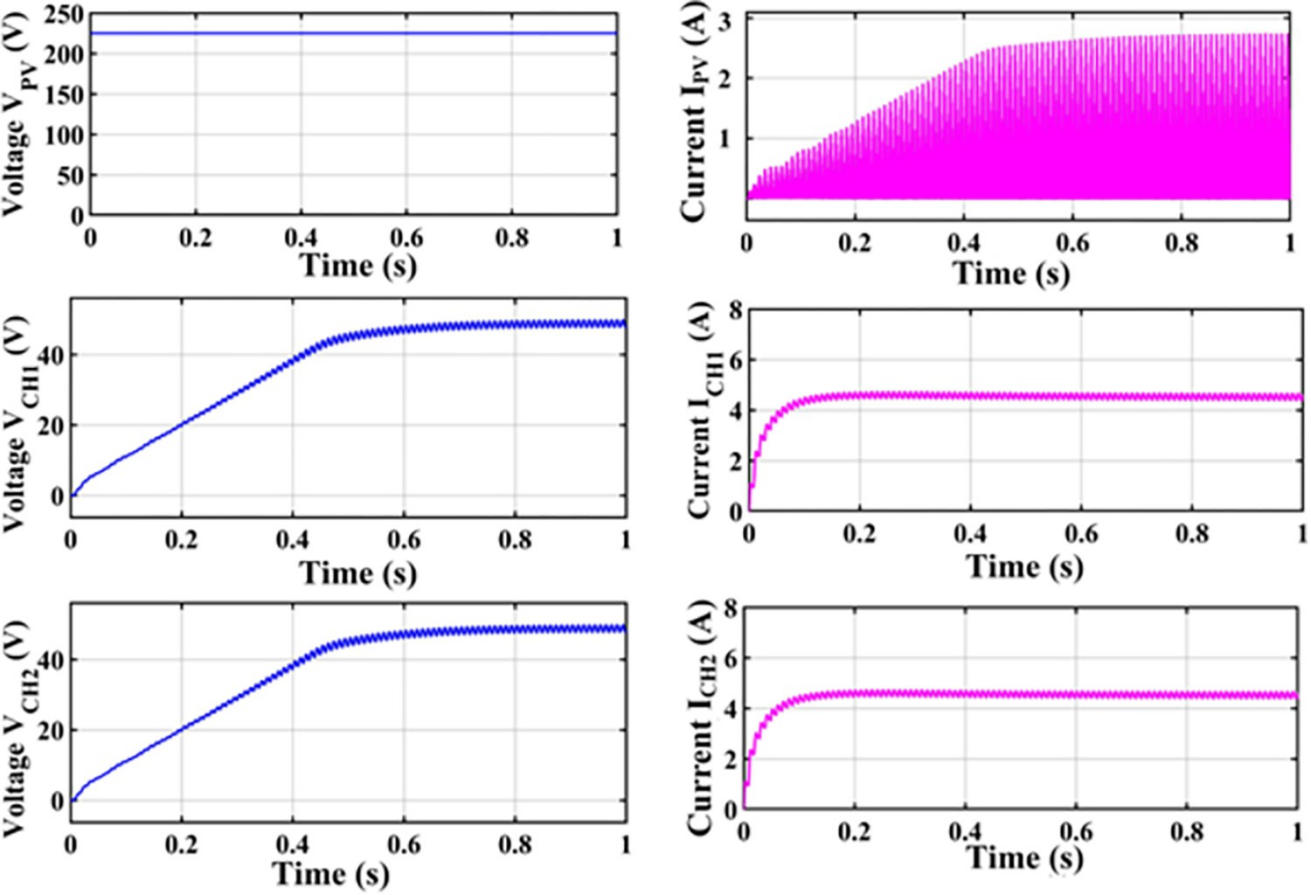
Mode II–PV to battery output waveforms.

**Fig 8:**
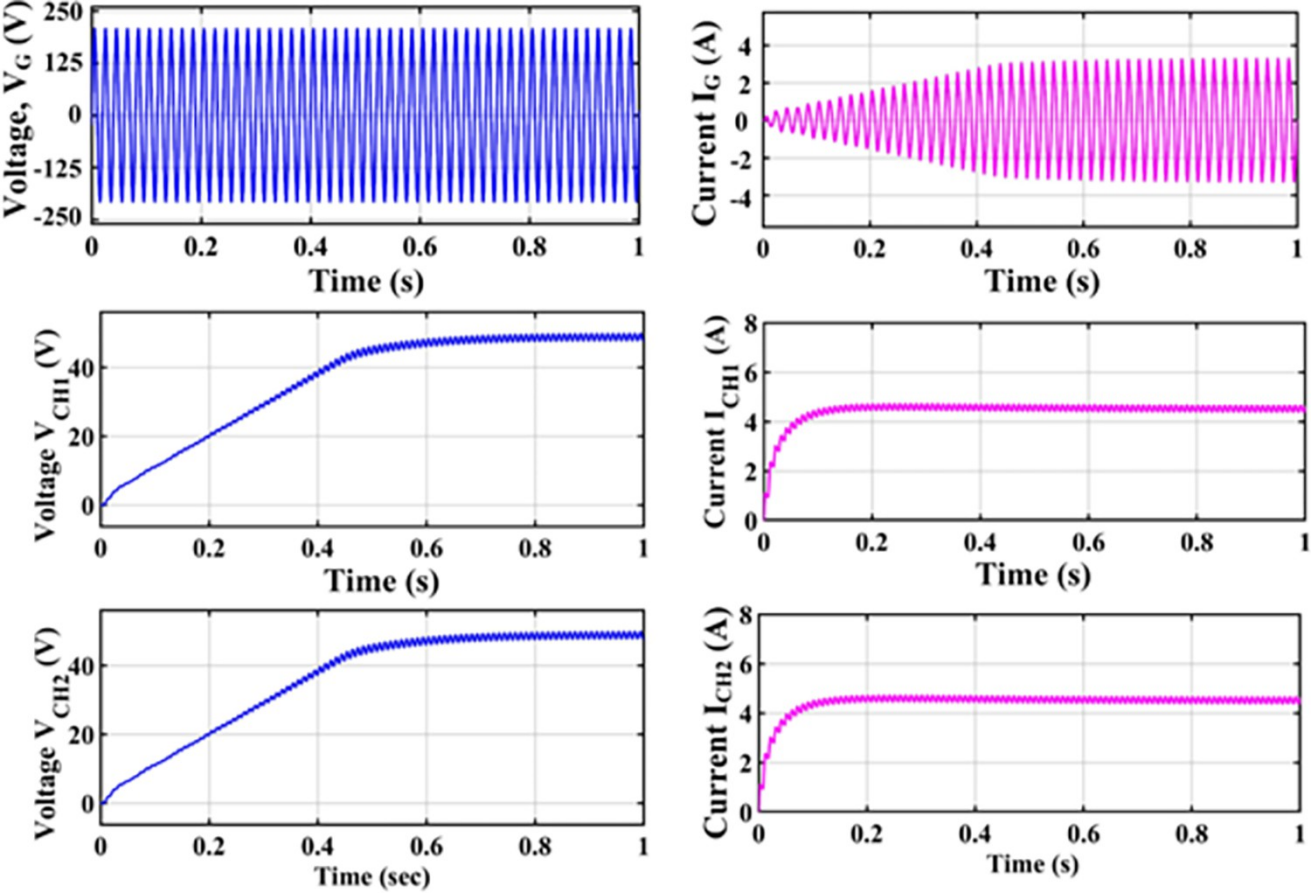
Mode III–Grid to battery output waveforms.

**Fig 9:**
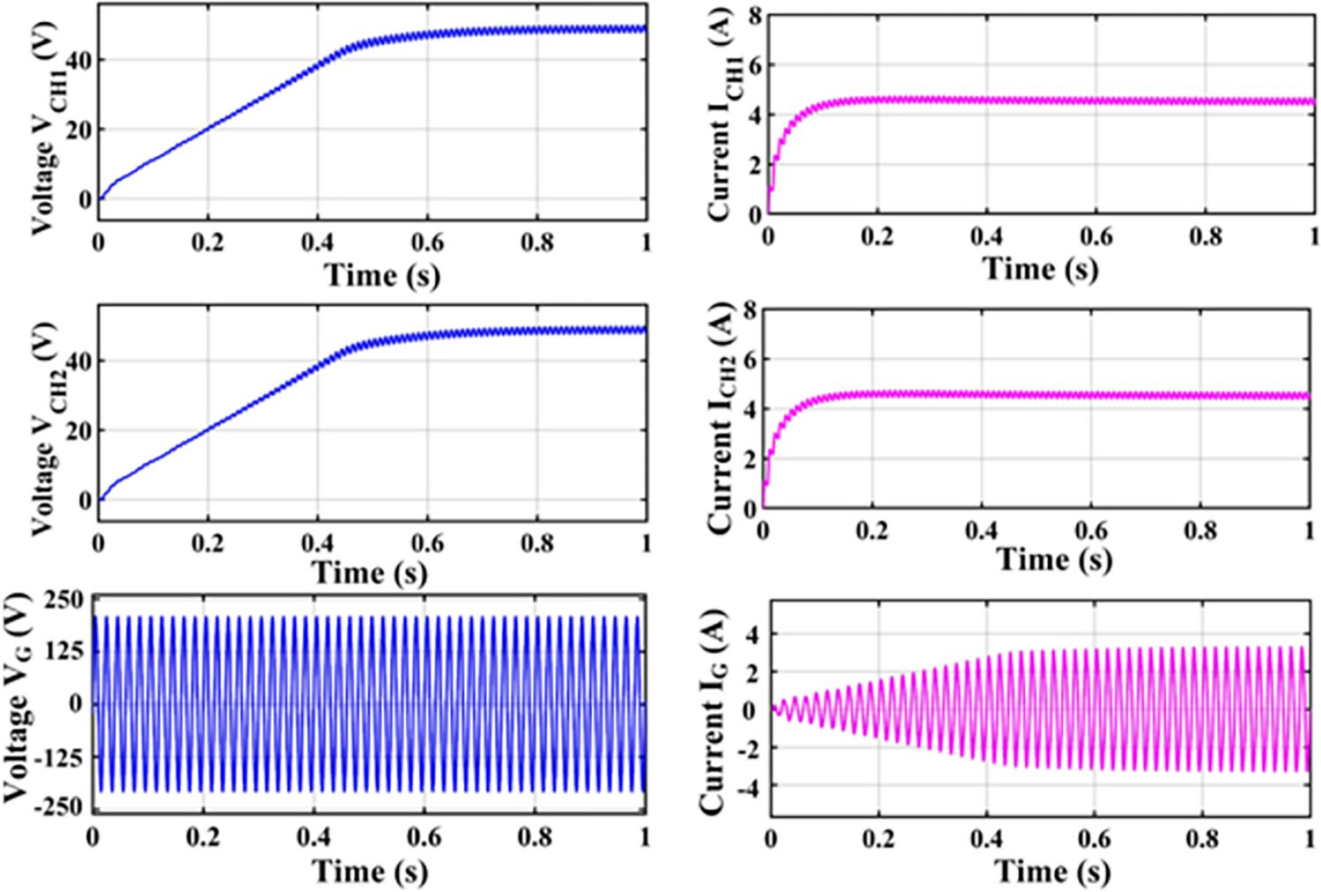
Mode IV–Battery to grid output waveforms.

**Fig 10:**
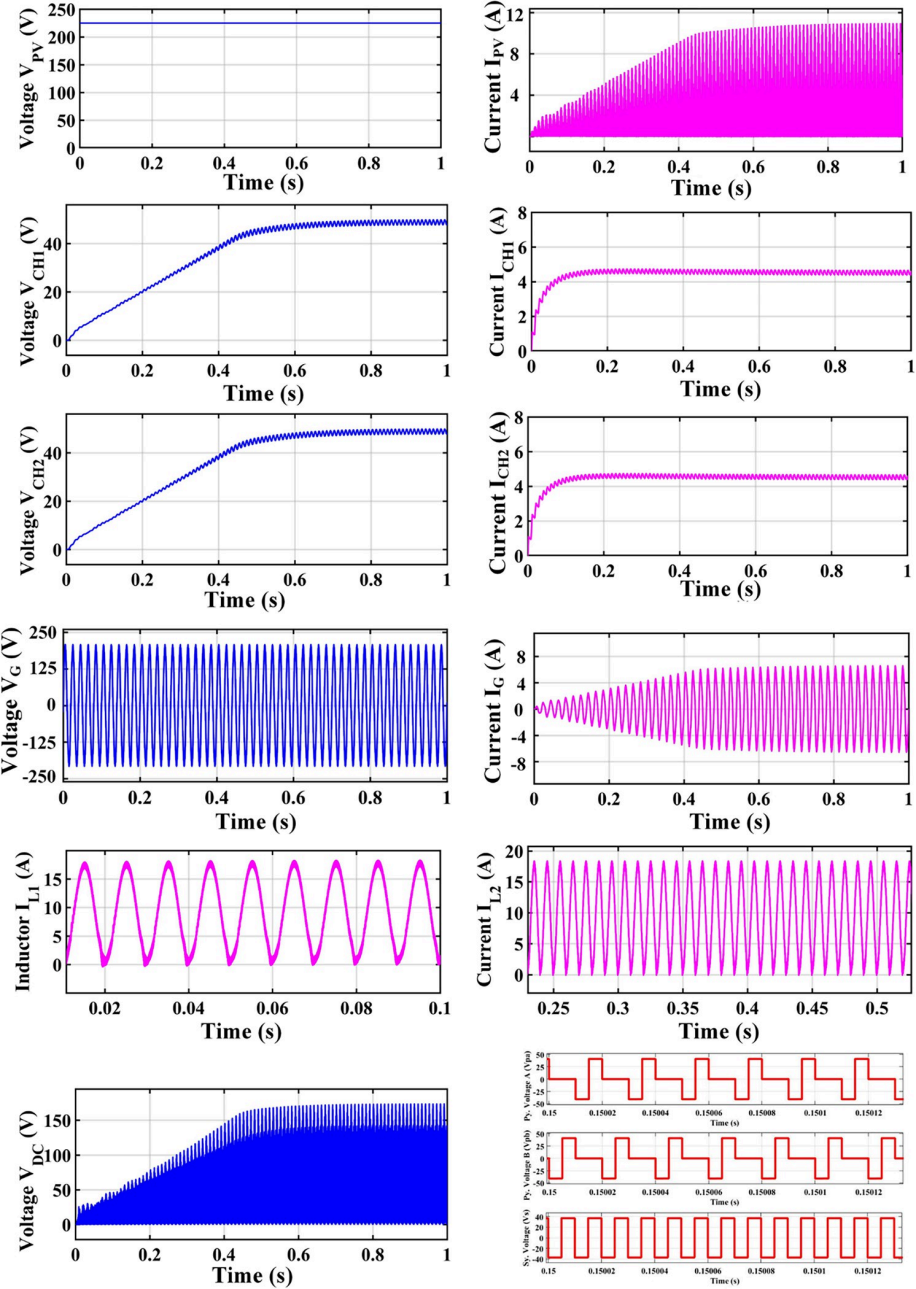
Mode V–PV to grid and battery output waveforms.

An H-bridge converter is connected between the MZSI and the grid. The capacitors used in the conventional ZSI is spilt into two equal capacitors for creating the multiport charger. A high frequency transformer is connected across these two capacitors. The HFT has the winding ratio of 2:1, where the primary side has two windings. Each windings are tapped from the two legs of capacitor for the creation of multiport as shown in simulation diagram. These two HFT are connected with the charger through the battery arrangement. This entire setup is been modelled as the simulation with the necessary calculations for capacitors, inductors, switching frequency and mode of operation using the Eqs (1)–(32). The specifications and the calculated parameters are given in Table 2. The simulation analysis is carried out validating the performance of proposed MZSI at the different mode of operations. The modes of operations are, PV to grid, PV to battery, gird to battery and battery to grid, and PV to grid/battery. Simultaneously the PV supplies the power to grid and charging station. So that, the simulation analysis and experimental analysis is carried out for totally five modes. The mode V is considered as the PV supplies power to the grid and the battery. The PWM is programed to regulate the switches as per this mode. In this mode, all seventeen switches used in this converter are in active condition. The switches in HBCs are operated with 50% of duty ratio and the MZSI operated with 25% duty ratio. The corresponding waveforms obtained for this mode is shown in Fig 6. The PV supplies nearly 500W power to the two charging stations (each 250W). Simultaneously, it supplies 1380W of power to the grid. Total power delivered to grid and charging stations are around 1880W with the efficiency of 90.18%. Similarly, the analysis is carried out for other five modes and the corresponding waveforms are shown in Fig 6 (PV to grid), Fig 7 (PV to battery), Fig 8 (Grid to battery), Fig 9 (battery to grid) and Fig 10 (PV to grid/battery). The simulation analysis is carried out for the five different modes and the efficiency of the proposed MZSI is validated. From the obtained results, it is observed that, the proposed MZSI has 90% of efficiency in all modes. This method has the enhanced performance as well as multiport charging option. The real time performance of the proposed MZSI is need to be validated for reducing the consequences of challenging factors in the practical applications.

**Table 2:** Proposed MZSI specifications (simulation and experiment).

Parameters	Value
Input voltage, *V*_in_	240 V
Grid voltage *V*_*g*_	230 V
Output voltage, *V*_*CH1*_	48V
Output voltage, *V*_*CH2*_	48V
Inductor value, *L*_1_ = *L*_2_	950 μH
Input capacitor, *C*_in_	220 μF
Filter Inductance, *L*_*f*_	7.6 mH
Switching frequency (MZSI), *F*_SW_	25 kHz
Switching frequency (HBC), *f*	50kHz
PV power output, *P*_PV_	2.1kW
Battery Rating, *P*_*B*_	500W
Battery Capacity	50Ah
Battery Voltage	48V
PV Open circuit voltage	264V
PV Short circuit current	10.09A

**Fig 11:**
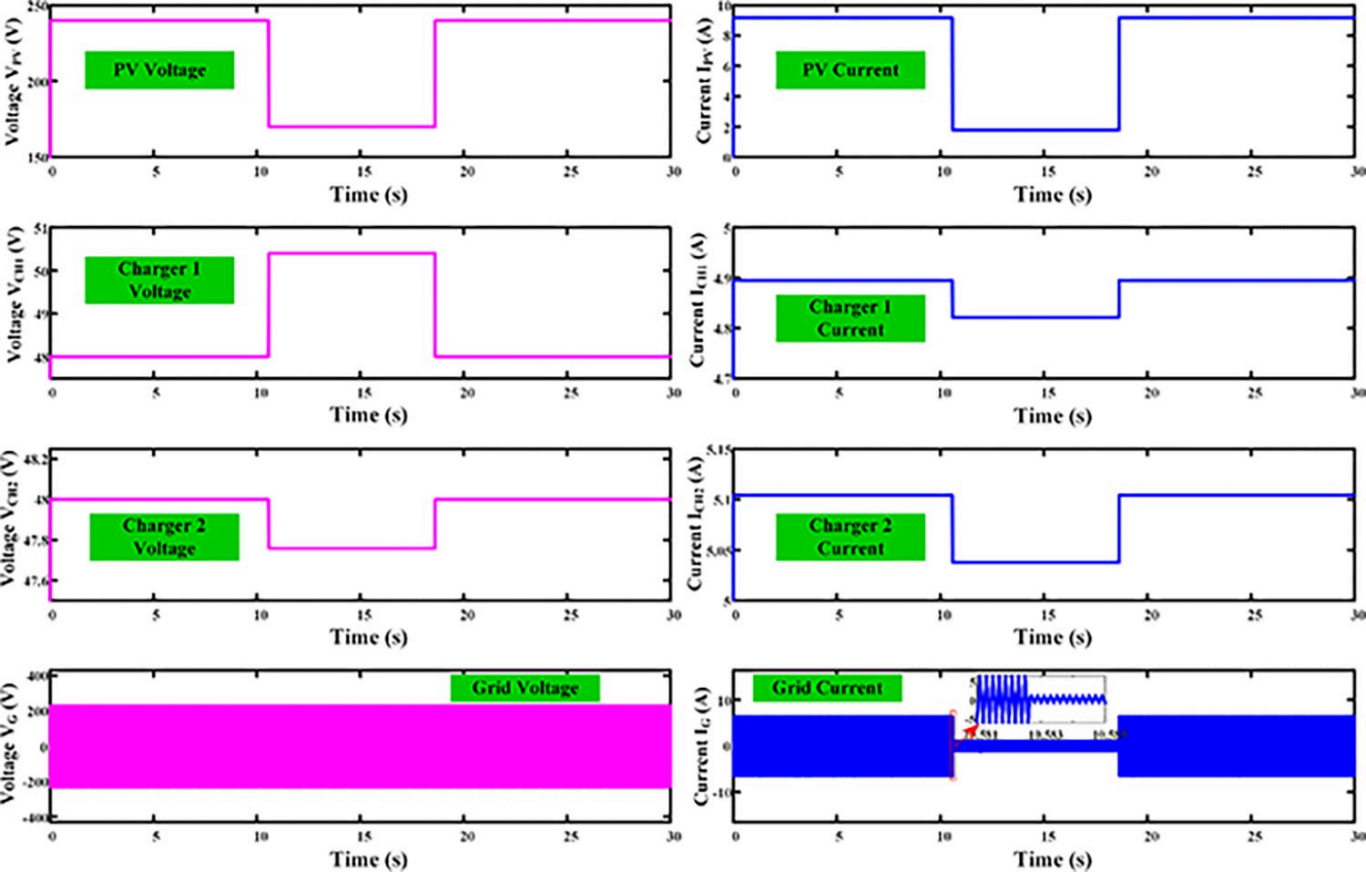
Mode V waveforms on dynamic conditions.

In first scenario (mode V (A)), PV generates rated power of 2200W power, and supplies 500W to charger side, and 1700W power to grid. When it was simulated in the MATLAB/Simulink®, the PV generates 2200W of power and 490W power to the chargers and 1480W power to the grid. In second scenario (mode V (B)), the PV system generates 300W of power which is not sufficient to meet out demand at charger side. In this mode, the PV supplies 300W to MZSI, and the required 200W power has been met by the grid. In this mode, grid supplies 240W of power to the MZSI. In third scenario (mode V (B)), the grid was operated in peak demand, where the grid experiences more stress. In this mode, the PV and grid supports the battery. To validate this mode, it is considered that, PV generates 1200W power and battery has 100% of SoC and it can able to supplies 500W for 3 hours. The MZSI will regulate the power from PV and battery to grid in this mode of operation. The dynamic operation between two states were observed where the PV was generated rated power generation of 2200W, and it delivers 1480W to the grid and 490W to the chargers. When the PV generation has been affected by the environmental conditions, it is considered that PV generates 300W, and charging station is on maximum demand. In this condition the grid supports the PV to supply the charging station that PV supplies 300W and grid supplies 240W. The transition between this two modes are shown in Fig 11.

The corresponding values of PV panel, dc link, grid, primary and secondary sides of HF transformer, Charger 1 and Charger 2 in all modes of operation are given in Table 3.

**Table 3:** Output of individual element in all modes of operation.

Parameters	Parameters	Mode 1	Mode II	Mode III	Mode IV	Mode V
PV	Voltage	240	240	240	240	240
	Current	8.52	2.25	0	0	9.16
HFT	Primary_1_ Voltage	40	40	40	40	40
	Primary_2_Voltage	40	40	40	40	40
	Secondary Voltage	40	40	40	40	40
DC-Link	Voltage	180	180	180	180	180
Grid	Voltage	230	230	230	230	230
	Current	8.56	0	2.34	2.1	6.4
Charger 1	Voltage	48.2	48.2	48.2	48.2	48.2
	Current	5.2	5.16	5.04	5.28	5.11
Charger 2	Voltage	48.6	48.6	48.6	48.6	48.6
	Current	4.92	5.13	4.99	5.21	5.07

The efficiency of this proposed MZSI is depends on the losses due to the semiconductor used in the circuit. The losses can be calculated by measuring RMS current and resistance of each component. The power loss across the inductor (I_L_) can be calculated using Eq (19),

$$P_{L}={I^{2}}_{L.RMS}R_{L}=\frac{{I^{2}}_{0}}{{\left(1-d_{4}\right)}^{2}}R_{L}$$ (19)

The conduction losses of the capacitor C is expressed as Eq (20),

$$P_{C}={I^{2}}_{C.RMS}R_{C}=\frac{{I^{2}}_{0}}{\left(1-d_{2}\right)}(2-d_{1}-d_{2}-d_{3}-d_{4})R_{c}$$ (20)

The losses that occurred in switches S_A_, S_B_, S_C,_ and S_D_ are given as Eq (21)–Eq (24), where R_1UP_, R_2LOW_, R_2UP,_ and R_2LOW_ are the resistances of the switches S_A_, S_B_, S_C_ and S_D_ when, they are in ON state.


$$P_{SA,RMS}={I^{2}}_{SA,RMS}R_{S}=\frac{{I^{2}}_{0}d_{1}}{{\left(1-d_{4}\right)}^{2}}R_{SA}$$ (21)

$$P_{SB,RMS}={I^{2}}_{SB,RMS}R_{S}=\frac{{I^{2}}_{0}d_{2}}{{\left(1-d_{4}\right)}^{2}}R_{SB}$$ (22)

$$P_{SC,RMS}={I^{2}}_{SC,RMS}R_{S}=\frac{{I^{2}}_{0}d_{3}}{{\left(1-d_{4}\right)}^{2}}R_{SC}$$ (23)

$$P_{SD,RMS}={I^{2}}_{SD,RMS}R_{S}=\frac{{I^{2}}_{0}d_{4}}{{\left(1-d_{4}\right)}^{2}}R_{SD}$$ (24)

The additional losses across each switches S_A_, S_B_, S_C_ and S_D_ were expressed as Eq (25),

$$P_{S,cond}=P_{SA}+P_{SB}+P_{SC}+P_{SD}=\frac{{{d_{1}I}^{2}}_{0}}{{\left(1-d_{4}\right)}^{2}}(d_{1}+d_{2}+d_{3}+d_{4})R_{S}$$ (25)

The switching losses of each switches can also be expressed as Eq (26)

$$P_{S,SW,Total}=\frac{1}{2}V_{S}I_{S}(t_{on}+t_{off})f_{s}$$ (26)

The overall switching losses of switches of S_A_, S_B_, S_C_ and S_D_ are

$$P_{S,sw,Overall}=P_{SA,SW}+P_{SB,SW}+P_{SC,SW}+P_{SD,SW}$$ (27)

Similarly, the losses across each diodes of D_a1_, D_a2_, D_a3_ and D_a4_ can be expressed as Eq (28),

$$P_{dcOverall}=[{I_{Da1rms}}^{2}+{I_{Da2rms}}^{2}+{I_{Da3rms}}^{2}+{I_{Da4rms}}^{2}]r_{d}$$ (28)

Eq (29) can be simplified and rewritten as Eq (30) as,

$$P_{dc}=[\frac{d_{m}2{I_{0}}^{2}}{{\left(1-d_{m}\right)}^{2}}+\frac{2{l_{0}}^{2}}{\left(1-d_{m}\right)}]r_{d}=[\frac{2{l_{0}}^{2}}{\left(1-d_{m}\right)}[\frac{d_{m}}{\left(1-d_{m}\right)}+1]]r_{d}$$ (29)

$$P_{dc}=\frac{2{l_{0}}^{2}}{{\left(1-d_{m}\right)}^{2}}r_{d}$$ (30)

Where r_d_ is the resistance of diode.

By combining all these losses of MZSI, the total losses in the circuit can be obtained. The power output and total efficiency of the proposed MZSI can be calculated from obtained overall losses (P_Overall loss)_ as given in Eq (31) and Eq (32)

$$P_{Out}=P_{in}-P_{overall\_losses}$$ (31)

$$\%Ƞ=\frac{P_{out}}{P_{out}+P_{overall\;losses}}100\%$$ (32)

The proposed MZSI is validated in a 500W two-port charging station and the measured losses for this specification are calculated. For this specification, the system experiences 4% of conduction loss, 2% of inductor loss, 1.5% of capacitor loss, and 0.6% of diode loss.

## 4. Experimental results

**Fig 12:**
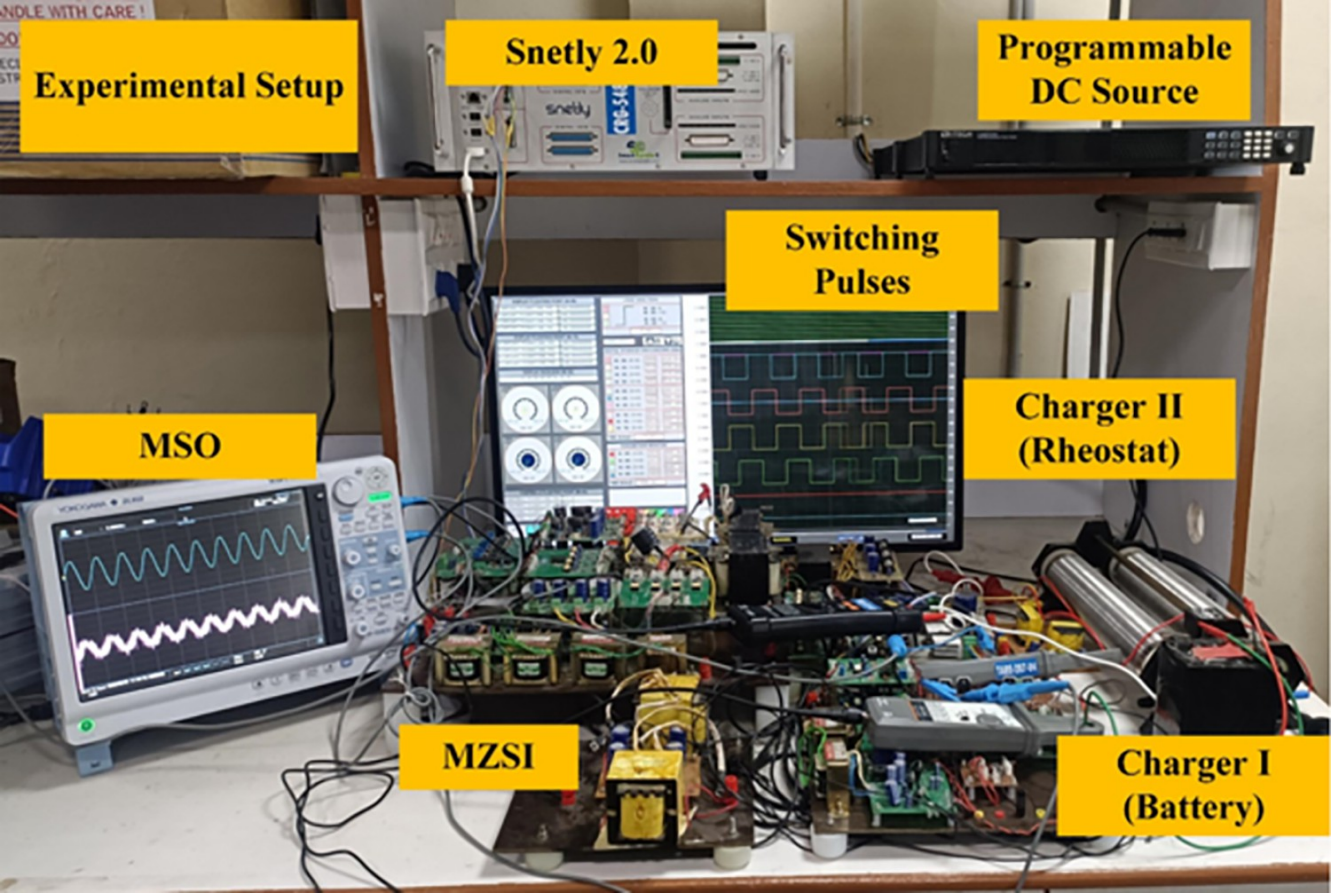
Experimental setup of the proposed system.

The proposed MZSI is been developed in real time as shown in Fig 12 to validate the performance of Mode V, i.e., power flow from PV to the battery and the grid. A 4 kW programmable bidirectional DC supply is used as the PV source. The designed battery charger requires 500W power to charge the two batteries simultaneously. In the simulation analysis, 2.2kW solar PV is been used, which can charge the two batteries and delivers 1.7kW power to the grid. In this mode, the experiment requires one power source and two loads. So, a programmable DC source is used as PV, battery and grid are used as loads. For the grid supply, the output terminal of HBC1 is connected to the grid through an AC distribution box. A mixed signal oscilloscope (YOKOGAWA DL950) is used for observing the output. In between the programmable DC source, battery, rheostat and grid, three HBC, two HFT and one MZSI were connected.

The MZSI is been constructed using four number of MOSFET (APT28M120L) switches, two inductors with 950μH, and four capacitors with 220μF as per the simulation model. The ratings of capacitors, inductors for the experiment is given in Table 1. Along with this inverter, three HBC were designed using the MOSFET switches. Each HBC has four number of MOSFET switches. The pulses for the switches are generated using Snetly 2.0. The pulses are generated through Snetly using the pulse width modulation technique. The generation of pulses are differed with respect to the operating modes. For the Mode V (PV to grid and battery), all three HBCs need to be operated, where each switches of the HBC are operated with the 50% of duty cycle. For the PV to grid operating mode, the HBC connected between MZSI and batteries are isolated, i.e., the Snetly will not give pulses to the switches. For the PV to battery operating mode, Snetly will not give pulses to the HBC switches in grid side. The Snetly is programmed for the generation of pulses with respect to each mode of operation. In the MZSI, the switches are operated with the 25% of duty cycle. And one more switch is been used in between the MZSI and PV. The purpose of this switch is protect the PV under reverse current. This switch will act as a normal blocking diode, also to isolate the PV when it requires to access the PV alone. These are the components used in the experimental setup for validating the proposed work.

**Fig 13:**
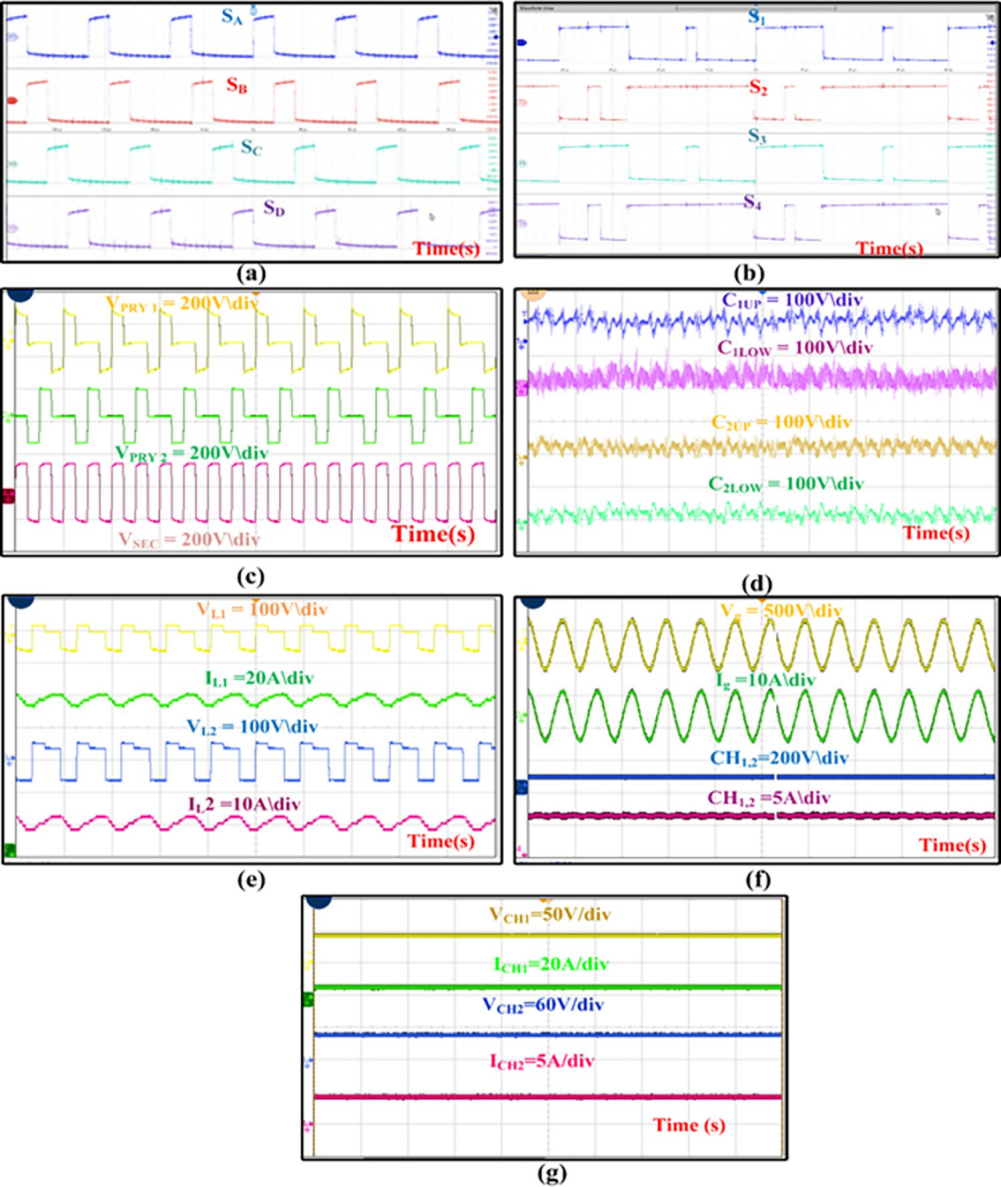
(a) H-Bridge Pulses (b) shoot through pulses (c)Voltage across transformer winding (d) Capacitor voltage (e)Inductor voltage and inductor current (f) Grid voltage and current (g) Charger output voltage and current.

For the Mode V, the PV will supply power to the grid and the battery. So that, all HBCs used in the experiment needs to be operated. The circuit connections between the components are given as per the connections in the simulation model. The programmable DC source is been programmed to supply 2200W power to the MZSI. It supplies the maximum current of 9.2A and maximum voltage of 240V to the MZSI. The switches between the PV and MZSI is been turned ON for the whole operating time. The switches in the MZSI are been operated with 25% duty cycle as shown in Fig 13. Shoot through condition of MZSI in each complete cycle, has the shoot through pulses as shown in Fig 13.

HFTs are taped between the capacitors for extracting voltage to the charging station. The HFTs connected with the batteries received the voltage of 45V in the two primary windings and 40V at the secondary side. This secondary voltage is fed to the HBCs connected with the batteries. As the HBCs are operated with the 50% duty cycles, it converts the 40V of AC into 48V of DC, which fed to the batteries. Similarly, the load terminal of MZSI is connected with the grid through the HBC. The MZSI has the link voltage V_PN_, and current output I_PN_ of 180V and 9.4 A. The grid voltage and grid current delivered from the HBC is shown in Fig 13, where it receives the voltage of 230V and current of 6.5A. Similarly, the voltage and current delivered to the batteries are shown in Fig 13.

Charger 1 receives the voltage of 48V and current of 5.3A, whereas the charger 2 receives the voltage of 48V and 4.8A current. The result and waveform for the Mode V is shown in Fig 13. Pulses for H-Bridge converters, shoot through Pulses, Transformer primary, secondary output, Capacitor voltage, Inductor voltage, Inductor current, Grid Voltage, Grid Current, Charger Output voltage and current wave forms for Charger were captured from the experimental setup.

In this mode of operation, the programmable DC source supplies 2200W of power output. After the conversion and tapping process, the charger 1 receives the power of 255W, charger 2 receives the power of 234W. Totally 489W of power has been delivered to the battery charging stations. Also, 1495W of power has been delivered to the grid. The MZSI receives 2200W of power output from the DC source and delivers 1984W of to the loads. From these values, the power efficiency has been calculated. The DC source power is been taken as the input power, and total power received at the battery and grid as taken as the output power. It gives the efficiency of 90.18%. From the theoretical calculations by considering switching loss, conduction loss and losses across capacitor and inductor gives the efficiency of 92%. The difference between the theoretical and measure efficiencies are around 1.6%. The proposed MZSI almost achieves the theoretical efficiency values.

In converter/inverter, the power conversion rate is an essential term to be considered, because it will be affected by various losses like switching loss, conduction loss and etc., The normal converter/inverter used between multiple sources like PV, battery, and the grid, will experiences more losses. This results in a reduction of power conversion efficiency. The proposed MZSI is operated between multiple sources, and, from the experimental results, it delivers power with 90.18% of efficiency. It has a higher efficiency rate, low switching losses, gives multiport charging options and it can be used everywhere. For the sustainable development of the EV charging stations, this MZSI can be the better solution. This setup can be easily interfaced with the existing charging stations.

## 5. Conclusion

In this work, an MZSI has been designed and developed for the multiport charging of EVs. The PV system is taken as the input source, and batteries and the grid are taken as the output. The MZSI is connected between PV-grid and PV-battery. The capacitor used in the conventional ZSI is split into two capacitors to enable the multiport option. Two HFTs are tapped across the capacitors for two charging ports. MOSFET switches are used to construct the inverters and converters, and these switches are operated with PWM technique using Snetly 2.0. This entire setup is modelled in MATLAB/Simulink® and was also constructed in the experimental setup. The proposed MZSI has been validated under different modes of operation, such as PV to battery and grid, PV to battery, PV to grid, grid to battery, and battery to grid. The performance of the MZSI under these modes has been observed and plotted as waveforms. The efficiency of the MZSI has been calculated, and it gives 90.18% efficiency in the operating condition of PV to grid and battery. Compared to the ZSI, the proposed MZSI has enhanced efficiency and provides multiport charging options. The construction cost is very similar to the conventional ZSI, whereas two additional capacitors, two HBCs, and two high-frequency transformers were used in the MZSI. Due to these additional components, the cost of the MZSI is 1.5 times greater than that of the normal ZSI. The proposed MZSI has many advantages over the normal ZSI in terms of better efficiency, simple control method, simple construction, and low cost. As the use of electric vehicles is growing in recent years, this proposed MZSI can be a better solution for EV charging stations.
